# Health-Related Behaviors in Swimming Pool Users: Influence of Knowledge of Regulations and Awareness of Health Risks

**DOI:** 10.3390/ijerph13050513

**Published:** 2016-05-19

**Authors:** Francesca Gallè, Laura Dallolio, Manfredo Marotta, Alessandra Raggi, Valeria Di Onofrio, Giorgio Liguori, Francesco Toni, Erica Leoni

**Affiliations:** 1 Department of Movement and Well-Being Sciences, University of Naples “Parthenope”, Via Medina 40, Naples 80133, Italy; francesca.galle@uniparthenope.it (F.G.); giorgio.liguori@uniparthenope.it (G.L.); 2 Department of Biomedical and Neuromotor Sciences, University of Bologna, Via S. Giacomo 12, Bologna 40126, Italy; alessandra.raggi2@studio.unibo.it (A.R.); erica.leoni@unibo.it (E.L.); 3 Local Health Unit of Romagna, Unit of Hygiene and Public Health, Via Coriano 38, Rimini 47854, Italy; manfredo.marotta@auslromagna.it (M.M.); francesco.toni@auslromagna.it (F.T.); 4 Department of Sciences and Technologies, University of Naples “Parthenope”, Business District, Block C4, Naples 80143, Italy; valeria.dionofrio@uniparthenope.it

**Keywords:** swimming pools, swimmer behaviors, knowledge, health risks, health promotion

## Abstract

*Background*: Swimming pool attendance exposes users to infection and chemical risks that could be largely reduced with the adoption of healthy behaviors. This study aims to investigate if the knowledge of swimming pool regulations and awareness of health risks can be associated with users’ health-related behaviors. *Methods*: A cross-sectional study was conducted using self-administered questionnaires to collect data from two different target groups of swimming users: 184 adults and 184 children/adolescents. The association between specific variables and patterns of behaviors and knowledge was assessed through multivariate logistic regression models. *Results*: Although more than 80% of both groups declared they knew the regulations, compliance with healthy behaviors was often unsatisfactory, especially in adolescents and youth. In the children/adolescents group, healthy behaviors significantly increased with the frequency of attendance per week. In both groups, compliance increased with educational level (of parents for children/adolescents), while no positive association was observed between viewing the regulations and adopting appropriate behaviors. In the adult group, a higher knowledge/awareness of health risks was related to decreased odds of at least one unhealthy behavior. *Conclusions*: Guaranteeing the public display of regulations in swimming facilities is not sufficient to promote and change health-related behaviors. Much more attention should be given to educational interventions aimed to increase knowledge of health risks and the awareness that bathers are directly responsible for their own well-being.

## 1. Introduction

Swimming and other water-based exercises are excellent ways to practice physical activity and to gain health benefits [1,2]. Swimming pools are used by a large variety of people, varying in age, health, and hygienic standards—not only to practice a sport, but also for recreational and educational use and rehabilitation therapy [3]. Furthermore, the ability to swim is important from a safety promotion perspective, as it contributes to a decrease in drowning accidents. In Italy, swimming is the third most popular sport, with approximately 3.9 million people involved in aquatic sports and about a million people attending swimming facilities for aqua gym, hydrotherapy, and other aquatic activities [4,5]. However, besides the health benefits derived from swimming and recreational water activities, the swimming pool environment exposes people to different health risks associated with microbial and chemical contamination or drowning and injury hazards. 

Infection risk in swimming pools is widely documented in the literature [6,7]. The users are the most important source of microorganisms of fecal origin, which may be washed from bathers’ bodies or accidentally released with feces or vomit into the pool water [8,9]. Human shedding of non-fecal microorganisms originating from the skin, hair, saliva, or mucus is another important means of transmission of pathogens and opportunistic microorganisms by contact not only with contaminated water, but also with surfaces of pool edges and shower floors [10,11,12].

Chemical risks are mainly associated with a variety of disinfection by-products (DBPs) deriving from the reaction of the chemicals used for disinfection (generally chlorine-based products) with the organic materials released by bathers (sweat, sebum, urine, saliva, cosmetics, sunscreens, *etc.*) [13,14,15,16]. The average release of anthropogenic pollutants into the pool water during 30 min of exercise is estimated in 250 mg non-purgeable organic carbon, 77.3 mg total nitrogen, 37.1 mg urea, and 10.1 mg ammonium per bather [17]. Although the exposure to DBPs in swimming pools has been associated with ocular and respiratory symptoms, and some of these products are genotoxic or cytotoxic in *in vitro* assays, studies exploring the relationship between swimming environments and adverse health effects due to DBPs draw an inconclusive picture, mainly due to the lack of large-scale prospective studies. Despite this, the management of aquatic facilities must aim to reduce DBPs, and users can play an active part in reducing water contamination. Personal hygiene and health-related behaviors are essential in reducing the spread of microorganisms and minimizing the introduction of DBP chemical precursors into the water [18,19,20,21]. 

Recommendations on personal hygiene and correct behaviors are provided by the Guidelines of the World Health Organization (WHO), while in 2001 the U.S. Centers for Diseases Control and Prevention (CDC) launched the “Healthy Swimming Program” which, through its “Triple A’s of Healthy Swimming: Awareness, Action, Advocacy”, provides information for the public, public health and medical professionals, and aquatics staff in order to maximize the health benefits of swimming and minimizing the risk of illness and injury [22]. In the category of “Awareness”, the Healthy Swimming Program includes the healthy behaviors of users. 

In Italy, in accordance with the National Guidelines [23], each swimming facility must have a regulation on public display showing personal hygiene measures and the correct behaviors to be respected. All bathers should be encouraged to use the toilets correctly before bathing and to take a pre-swim shower before entering the swimming pool, since a simple shower removes most pollutants just within the first 60 s [24]. The use of a footbath before entering the swimming area is also recommended, as well as the use of a swimming cap and footwear, while some unhygienic habits must be avoided—for example, the voluntary release of organic fluids or secretions into the water. 

These measures represent an important tool in the control of health risks in swimming pools, and are just as important as the monitoring of microbial and chemical quality of the water and the management of the plants, aspects that are taken into account much more in the literature [25,26,27,28,29,30]. On the contrary, few studies have been performed to assess the compliance of swimmers to hygiene-related rules and their knowledge of the risks associated with bathing in swimming pools. To the best of our knowledge, reports are available from a telephone survey of the Water Quality & Health Council (WQHC) in the U.S. [31,32] and from a few investigations that were performed in different Italian cities [33,34,35].

This study investigates the behavior of swimming pool users, their knowledge of swimming pool regulations, and their awareness of the health risks associated with exposure to the swimming environment. Its aim is to investigate the compliance of swimming pool users to healthy behaviors and how their habits change in relation to the declared knowledge of swimming pool regulations and the awareness of health risks, aside from some other individual variables (age, gender, educational level). 

## 2. Materials and Methods

### 2.1. Study Design

From June to August 2015, a cross-sectional survey was carried out in six swimming pools in the province of Rimini (Emilia Romagna Region, Italy). All the pools, whether owned publicly (five) or privately (one), were open to the general public and were used mainly by the residents in the area. Two contained both indoor and outdoor pools, while the remaining four had just an indoor pool. Two different anonymous self-administered questionnaires were distributed to the users (adults and minors) who regularly attended the swimming facilities (at least once a week), after giving their informed consent. Occasional users, being very few, were excluded because they were unrepresentative. The questionnaire designed for adults was given to those aged ≥18 years (henceforth adults), and the one for minors (henceforth children/adolescents) was filled out by their parents. A total of 600 questionnaires were distributed.

### 2.2. Questionnaires

For the adult users, the questionnaire previously described by Liguori *et al.* [36] was used, including 38 items divided into three sections: the first section collects personal data (age, gender, residence, education) and a description of activities carried out in swimming pools (items 1–8); the second section inquires about the behaviors while the user attends swimming pools (items 9–26); the third section investigates if the user knows the swimming pool regulations and the rationale behind them, and if he/she is aware of the health risks associated with swimming pools.

For the children/adolescents, a preliminary version of the questionnaire was drafted and tested on a pilot sample of 30 parents of children/adolescents who attended the swimming pools. The participants were then asked for feedback to identify ambiguous and difficult questions, which were re-worded. The subjects involved in the pilot study were excluded from the cross sectional study. The final version includes 21 items organized into four sections: the first section collects personal data (age, gender, residence, parent’s educational level), previous/current attendance of swimming pool and number of days and hours per week (items 1–6); the second section inquires about the behaviors while attending swimming pools (items 7–18); the third section investigates if the child/adolescent has read the swimming pool regulations or has been informed of the rules to follow (items 19–20). In the last part of the questionnaire (item 21) the parent is asked to declare if the child/adolescent has ever shown symptoms of illness after using a swimming pool (disorders of the upper respiratory tract, skin, eyes or ears, abdominal pains, vomit, diarrhea) and/or undergone traumas due to trips, slips, or falls. Since the questionnaires were anonymous and self-completed, ethical approval was not required, according to the Italian regulations.

### 2.3. Statistical Analysis

The questionnaire responses were entered into a Microsoft Excel spreadsheet and analyzed using SPSS Statistics Version 22 for Windows (IBM, Chicago, IL, USA). A descriptive analysis was undertaken to evaluate demographic and behavioral information and knowledge of regulations reported by parents of children/adolescents and by adults. Chi-squared test was used to assess significant differences between the two target groups and to analyze age differences. Five different unhealthy habits (lack of pre-bathing shower and footbath, no use of proper footwear and swimming cap, and consumption of food in swimming pool environment) were used to create a new variable, defined as the adoption of at least one out of five unhealthy behaviors. Levels of knowledge shown by adults about health risks and health problems suffered by children/adolescents as a consequence of attending swimming pools were also evaluated by analyzing the frequencies of answers.

Subsequently, univariate and multivariate analyses were performed to determine possible associations among the collected data. In the adults group, the adoption of at least one unhealthy behavior was considered as an outcome, while gender, age, educational level, knowledge of regulations, and level of knowledge/awareness of health risks were considered as independent variables. The level of knowledge/awareness of health risks was categorized into six classes, with a score from 1 to 6, defined by the number of correct answers (from 1 to 6) to the specific questions about the knowledge of healthy behaviors and awareness of health risks. 

In the sample of children/adolescents, the adoption of unhealthy behaviors (outcome 1) and the report of diseases (outcome 2) were considered as dependent variables, while gender, age, parent’s educational level, knowledge of facility regulations, frequency of attendance per week (categorized as ≤1 h, 2–3 h, 4–5 h, and ≥6 h per week) were considered as independent variables. The explanatory variables were selected by backwards selection. Odds Ratios (ORs) and 95% Confidence Interval (CI) were reported. ORs were weighted for gender and age. The significance level chosen for all analyses was 0.05.

## 3. Results

### 3.1. Compliance to Healthy Behaviors

Out of a total of 372 questionnaires collected (about 62% response rate), 368 (184 from parents and 184 from adult users) were correctly filled out. 

Table 1 summarizes the personal data and the main declared behaviors assumed by children/adolescents and adults in swimming pools. Both samples of swimming pool users had a higher proportion of females (children/adolescents: 53.3% females, 46.7% males; adults: 58.2% females, 41.8% males). The most common educational level among parents was a university degree (35.9%), while among adults the high school diploma was most represented (45.3%). Although a great part (more than 80%) of both groups declared they knew the hygienic rules of the facility, healthy behaviors appear to be highly performed by the group of children/adolescents, with significant differences regarding pre-swim shower (*p* = 0.001), footbath before entering in the swimming area (*p* < 0.01), and use of proper footwear (*p* < 0.05). However, the behaviors are not homogeneous within the same target group. Considering children/adolescents and adults together and stratifying by age (Table 2), the groups most frequently presenting at least one unhealthy behavior are the central age groups, the adolescents (14–17 years) in the sample of children/adolescents, and the young adults (18–39 years) in the sample of adults. Significant differences by age group are confirmed for pre-swim shower and use of proper footwear (*p* < 0.05).

Regarding the questions addressed only to adults concerning other healthy behaviors, 80.8% do not exchange personal objects with other people, 87.1% do not use cosmetics before entering the pool, and 91.5% and 86.5% do not use glasses or contact lenses, respectively. However, quite substantial percentages of adults report at least one episode of urination or clearing their nose/mouth inside the pool (16.3% and 34.5% respectively). 

### 3.2. Knowledge and Awareness

Adults were also asked about their knowledge of correct behaviors (five items) and their awareness of the infectious risks associated with swimming pool environments (one item) (Table 3). A percentage of users varying between 47% (time between meals and bathing) and 80% (reasons for the use of swimming cap) correctly know the reasons for healthy behaviors, while knowledge of the principal agents of skin infections is above 85%. Only 2.1% (4 out of 184 respondents) users gave correct answers to all six questions of Table 3. 

Parents were also asked about symptoms and morbidities suffered by their children/adolescents, in relation to the attendance of swimming pool environments. Among the symptoms most frequently referred by parents, rhinitis was reported by 11.9% and eye complaints by 27.2%. Skin infections (*mycosis* or *verrucas*) are reported by 8.1% of respondents, while the most frequent skin disorder is itch, which affects around a third of participants (32.6%) (Table 4).

### 3.3. Multivariate Analysis

In the sample of adults, the multivariate logistic regression model shows a significant positive association (*p* < 0.05) only between the adoption of at least one unhealthy behavior and age group (Table 5). Being female or a young adult increases the probability of adopting at least one unhealthy behavior, as does having a low level of education. In general, a lower knowledge and awareness of health risks, measured with a score from 1 to 6 (number of correct answers to the questions of Table 3), results in a higher OR (an increased probability of adopting at least one unhealthy behavior), while having read the regulations does not appear to be related to the adoption of correct behaviors. 

In the sample of children/adolescents, the adoption of at least one unhealthy behavior (outcome 1) is significantly associated with the number of weekly hours of attendance, with a reduction of unhealthy behaviors in proportion to the increase in weekly hours (*p* < 0.05). The probability of having some unhygienic habits increases with age and is higher among males, while it decreases with the level of parents’ education. As in the adult group, the reported knowledge of the facility regulations does not show association with the adoption of correct behaviors. The report of symptoms and morbidities (outcome 2) is significantly associated with gender: females, who in this group adopt unhealthy behaviors (outcome 1) less than males, appear less prone to diseases (outcome 2), according to the reports of parents (Table 6). The risk of disease increases among adolescents, while it decreases in proportion the parents’ level of education and the declared knowledge of the rules. With the increase in weekly attendance, a reduction can be observed in reported diseases, together with a decrease in unhealthy behaviors (outcome 1). 

The wide range of confidence intervals registered in both multivariate analyses could be attributed to the small size of each subgroup. 

## 4. Discussion

In accordance with the Italian guidelines, swimming facilities must display regulations specifying the correct behaviors to adopt in the pool environment. In our sample, more than 80% of respondents said they had read the rules (adults) or that they were informed of them (children/adolescents). The percentages are higher than those reported in similar investigations carried out in other Italian towns, which range from 48% to 75% [34,35]. Having read or being aware of the regulations does not, in itself, guarantee that basic protective habits are adopted. Indeed, our findings show that the simple knowledge of the rules is not associated with the compliance to healthy behaviors either in children/adolescents or in adults, who instead tend to adopt appropriate behaviors in relation to their knowledge/awareness of the health risks.

Adhesion to the rules seems related with the age. The age groups 14–17 and 18–39 show the worst compliance, as observed in other studies [35]. Around a third of those belonging to these age groups present at least one unhealthy behavior. A typical characteristic of adolescents and youth is the tendency to refuse rules and to escape the control of their parents, a problem not arising in the case of younger children. The children/adolescents who attend the pool more frequently show a greater respect for hygienic norms: attending the facility on a regular basis therefore appears to encourage younger users to respect the rules, and this may also be due to the presence of a trainer in the courses held for this age group.

The behaviors that show the lowest compliance are the pre-swim shower (86% of children/adolescents and 69% of adults) and the use of the footbath (85% of children/adolescents and 69% of adults). Although the compliance of 69% for pre-swim shower in adults may appear low, it is nevertheless higher than what was observed in the United States in a survey carried out by the Water Quality and Health Council in 2012, which reports compliance in just 57% of respondents [32]. Pre-swim showering reduces the number of microorganisms, sweat, cosmetics, and other organic compounds which are precursors of DBPs. It therefore helps not only to decrease the risk of infection, but also the risks deriving from chemical contamination—in particular, the potential allergic and irritant effects of DBPs on the upper respiratory tracts and skin [18,37]. A worrying fact emerging from our study is that around a third of the sample of children/adolescents suffer from itchy skin, a symptom often associated with irritative or allergic phenomena. The use of the footbath before entering the swimming area is recommended by WHO guidelines, which, in particular, suggest a strategic placement of footbaths in order to minimize the transfer of dirt into the pool [6]. The observed compliance of 69% in adults, a percentage comparable to those observed in other Italian cities by Pasquarella *et al.* [35,38], is unsatisfactory and needs to be improved. The other healthy behaviors (use of proper footwear and swimming cap, no consumption of food, no use of cosmetics or personal objects such as glasses and jewelry in the water) show a high level of compliance, around 90% in both samples. However, the fact remains that 16.3% of adults report at least one episode of urination inside the pool, a percentage within the range of 5%–30% observed in other Italian surveys [33,35] and lower than the percentage reported by the WQHC in the United States (19%).

In the sample of children/adolescents, the frequency of symptoms and illnesses is lower in females (significant differences: *p* = 0.01) and decreases in proportion to the number of weekly hours attended (*p* = 0.02); that is to say, in the users (females and high frequency bathers) who also present a greater compliance with healthy behaviors.

Some limitations of this study should be considered. The first concerns the selection of the sample, since only regular swimming pool users were included in the survey. It was not possible to collect the data for the children/adolescents who attended the swimming pool alone, without parents giving informed consent. Furthermore, the respondents had a medium/high education background, which could be different from users who did not participate in the survey (62% response rate). For this reason, it is possible that this study underestimates the prevalence of inappropriate behaviors. Another limitation is the small size of the samples, which resulted in a wide range of confidence intervals when multivariate analysis was performed to relate unhealthy behaviors with the different variables. In addition, the use of different questionnaires has not allowed comparison of the two groups (children/adolescents and adults) for all the variables.

Despite these limitations, our results contribute to the knowledge of such behaviors and, together with those obtained in other Italian cities, allow us to identify the direction in which preventive interventions should be oriented. There is a large and growing literature on the effectiveness of health information on behavior change [39,40,41]. Traditional written information and advice-giving can improve knowledge, but they are rarely sufficient to change behavior. Education programs should be aimed at raising awareness, taking into account the numerous complex influences on behavior change. In accordance with the theoretical basis of the Health Belief Model [42,43], perceived risk is an important predictor for assuming protective behaviors. Swimming pool users are therefore more likely to be motivated to adopt prevention strategies if they perceive the health risk and are aware of the seriousness of the morbidities and accidents associated with swimming pool attendance.

## 5. Conclusions

Since correct behaviors of users seem to be related to their awareness of health risks, more attention should be given to health promotion in swimming facilities. As recommended by the WHO [6], all stakeholders (facility workers, local authorities, public health operators, families, schools, general practitioners) could play an important role in minimizing unhealthy behaviors and ensuring pool safety through public education and appropriate and targeted information. The provision of generic information and the display of regulations are not sufficient to change behaviors. Educational interventions should aim to increase the knowledge/awareness of health risks and the awareness that swimming pool users are directly responsible for their own well-being by practicing good hygiene. According to a multi-action approach, a key role could be played by trainers and bathing assistants who should be properly trained not only in technical skills but also in educational and preventive aspects. Ideally, a network should be established between the structures responsible for public health and the managers of sports facilities, aimed at the promotion of health through information to clients, training of staff and educational projects that take into account the needs of different target groups of swimming pool users, in terms of age and frequency of exposure, factors that our study has shown to be directly associated with behaviors.

## Figures and Tables

**Table 1:** Demographic data and declared behaviors of the two groups of bathers with corresponding *p* values.

Variables	Answers	Children/Adolescents	Adults	*p*-Value
		n (%)	n (%)	
**Number of respondents**		184	184	
**Mean age ± SD**		7.9 ± 2.9 years	43 ± 14.1 years	
**Gender**	Males	84 (46.7)	77 (41.8)	0.39
	Females	96 (53.3)	107 (58.2)	
**Knowledge of swimming pool regulations**	Yes	148 (82.7)	147 (80.3)	0.56
	No	31 (17.3)	36 (19.7)	
**Pre-swim shower**	Always	159 (86.4)	119 (69.2)	
	Almost always	14 (7.6)	29 (16.9)	<0.01
	Sometimes	7 (3.8)	10 (5.8)	
	Never	4 (2.2)	14 (8.1)	
**Footbath before entering the swimming area**	Always	156 (85.2)	118 (69.4)	
	Almost always	13 (7.1)	22 (12.9)	<0.01
	Sometimes	9 (4.9)	13 (7.6)	
	Never	5 (2.7)	17 (10.0)	
**Use of proper footwear**	Always	178 (96.7)	155 (90.1)	
	Almost always	3 (1.6)	3 (1.7)	0.04
	Sometimes	1 (0.5)	7 (4.1)	
	Never	2 (1.1)	7 (4.1)	
**Use of swimming cap**	Always	176 (96.7)	164 (95.9)	
	Almost always	1 (0.6)	3 (1.8)	0.46
	Sometimes	0 (0.0)	1 (0.6)	
	Never	5 (2.8)	3 (1.8)	
**Use of swimming goggles**	Always	111 (61.7)	97 (57.7)	
	Almost always	20 (11.1)	15 (8.9)	0.58
	Sometimes	16 (8.9)	21 (12.5)	
	Never	33 (18.3)	35 (20.8)	
**Consumption of food in changing rooms**	Always	11 (6.0)	4 (2.3)	
	Almost always	1 (0.5)	2 (1.2)	0.14
	Sometimes	7 (3.8)	13 (7.6)	
	Never	165 (89.7)	153 (89)	

**Table 2:** Healthy behaviors adopted by participants in swimming pools by age group with corresponding *p* values.

Behaviors	Answers		Age Group (Years)						*p*-Value
			≤9	10–13	14–17	18–39	40–54	≥55	
**Pre-swim shower**	Always/	n	122	35	8	53	53	29	0.03
	Almost always	%	97.6	85.4	88.9	85.5	86.9	93.5	
	Sometimes/	n	3	6	1	9	8	2	
	Never	%	2.4	14.6	11.1	14.5	13.1	6.5	
**Use of proper footwear**	Always/	n	124	40	9	55	58	30	0.02
	Almost always	%	99.2	97.6	100	88.7	95.1	96.8	
	Sometimes/	n	1	1	0	7	3	1	
	Never	%	0.8	2.4	0	11.3	4.9	3.2	
**Footbath before entering the swimming area**	Always/	n	117	37	7	50	52	27	0.11
	Almost always	%	93.6	92.5	77.8	80.6	86.7	87.1	
	Sometimes/	n	8	3	2	12	8	4	
	Never	%	6.4	7.5	22.2	19.4	13.3	12.9	
**Use of swimming cap**	Always/	n	120	38	9	61	58	30	0.57
	Almost always	%	97.6	95	100	100	95.1	96.8	
	Sometimes/	n	3	2	0	0	3	1	
	Never	%	2.4	5	0	0	4.9	3.2	
**Consumption of food in changing rooms**	Always/	n	8	3	1	3	3	0	0.71
	Almost always	%	6.4	7.3	11.1	4.8	4.9	0	
	Sometimes/	n	117	38	8	59	58	31	
	Never	%	93.6	92.7	88.9	95.2	95.1	100	
**At least one /five unhealthy behaviors**	No	n	107	30	6	44	49	31	0.06
		%	85.6	73.2	66.7	68.8	74.2	86.1	
	Yes	n	18	11	3	20	17	5	
		%	14.4	26.8	33.3	31.3	25.8	13.9	

**Table 3:** Knowledge of health risks related to the attendance of swimming pools reported by adults (*n* = 184).

Adults’ Knowledge and Awareness	Answers	n (%)
**Time interval between meals and bathing**	1 h	6 (3.4)
	2 h ^a^	84 (46.9)
	4 h	65 (36.3)
	No time	24 (13.4)
**Reasons for the pre-swim shower**	Get used to water temperature	41 (22.8)
	Personal hygiene	128 (71.1)
	Get used to water temperature and personal hygiene ^a^	11 (6.1)
**Reasons for the use of swimming cap**	Hygiene/to avoid the release of hair into water ^a^	144 (80.0)
	Other reasons Must also be used by bald persons	36 (20.0)44 (29.7)
**Band-aid as adequate protection for wounds during bathing**	Yes	16 (8.8)
	No ^a^	139 (76.4)
	Do not know	27 (14.8)
**Individuals with infectious skin diseases can enter pool**	Yes	16 (8.8)
	No ^a^	140 (76.9)
	Do not know	26 (14.3)
**Knowledge of infections transmitted in pools ^b^**	*Mycosis*	158 (85.8)
	*Verrucas*	173 (94.0)
	Other	33 (18.1)
	Do not know any infections	8 (4.4)

**^a^** answers considered correct; **^b^** the knowledge of at least an infection was considered a correct answer.

**Table 4:** Symptoms and morbidities suffered by children/adolescents related to the attendance of swimming pools (*n* = 184).

Reported Symptoms and Morbidities		Children/Adolescents
		n (%)
**Upper respiratory tract symptoms**	Yes	50 (28.2)
	Sore throat	5 (2.8)
	Dry mucus	24 (13.6)
	Stuffy and runny nose	21 (11.9)
**Eye symptoms**	Yes	48 (27.2)
	Irritation	35 (19.8)
	Lacrimation	8 (4.6)
	Other	5 (2.8)
**Cutaneous diseases**	Yes	19 (10.9)
	*Mycosis*	3 (1.7)
	*Verrucas*	11 (6.4)
	*Urticaria*	4 (2.3)
**Ear diseases**		8 (4.5)
**Gastroenteritis symptoms**		4 (2.3)
**Itch**		56 (32.6)
**Injuries**		9 (5.0)

**Table 5:** Results of the logistic regression model applied to the adult sample (*n* = 147).

Explanatory Variable		Outcome: At Least One Unhealthy Behavior	*p*-Value
		OR (IC 95%)	
**Gender ^a^**	Males	*reference*	0.30
	Females	1.32 (0.59–2.92)	
**Age group ^a^**	18–39	*reference*	0.02
	40–54	0.73 (0.33–1.61)	
	≥55	0.26 (0.07–0.90)	
**Educational level ^a^**	Primary/middle school	*reference*	0.09
	High school	0.26 (0.03–2.07)	
	University degree	0.44 (0.05–3.75)	
**Have read the facility regulations ^a^**	Yes	*reference*	0.57
	No	0.77 (0.29–2.02)	
**Level of knowledge and awareness of health risk, score ^b^**	1	1.63 (0.07–36.72)	0.62
	2	2.29 (0.23–22.14)	
	3	0.97 (0.11–8.61)	
	4	1.32 (0.16–10.91)	
	5	1.43 (0.15–13.33)	
	6	*reference*	

**^a^** Not included in the best fitting logistic regression model; **^b^** Included in the best fitting logistic regression model.

**Table 6:** Results of the logistic regression model applied to the children/adolescents sample (*n* = 152).

Explanatory Variable		Outcome 1: At Least One Unhealthy Behavior	*p*-Value	Outcome 2: Reported Symptoms and Morbidities	*p*-Value
		OR (IC 95%)		OR (IC 95%)	
**Gender ^a,b^**	Males	*reference*	0.31	*reference*	0.01
	Females	0.87 (0.35–2.14)		0.11 (0.01–1.29)	
**Age class ^a,b^**	≤9	*reference*	0.10	*reference*	0.72
	10–13	2.68 (1.02–7.06)		0	
	14–17	5.55 (0.80–38.29)		58.1 (2.50–1350.15)	
**Parent’s educational level ^a,b^**	Primary/middle school	*reference*	0.31	*reference*	0.40
	High school	0.30 (0.11–0.76)		0.31 (0.05–1.99)	
	University degree	0.57 (0.17–1.91)		0	
**Have read or have been informed of the facility regulations ^b,c^**	Yes	*reference*	0.23	*reference*	0.64
	No	0.81 (0.27–2.43)		2.22 (0.38–12.79)	
**Attendance per week (hours) ^b,c^**	≤1	*reference*	0.02	*reference*	0.53
	2–3	0.39 (0.17–0.86)		0.09 (0.02–0.43)	
	4–5	0.11 (0.02–0.57)		0.15 (0.01–1.63)	
	≥6	0.12 (0.02–0.63)		0.08 (0.004–1.72)	
**At least one unhealthy behavior ^b^**	Yes			1.02 (0.19–5.58)	0.41
	No			1.02 (0.19–5.58)	

**^a^** Not included in the best fitting logistic regression model for outcome 1; **^b^** Not included in the best fitting logistic regression model for outcome 2; **^c^** Included in the best fitting logistic regression model for outcome 1.

## Abbreviations

The following abbreviations are used in this manuscript:

DBPs	Disinfection by-Products
CDC	Centers for Diseases Control and Prevention
WHO	World Health Organization
WQHC	Water Quality & Health Council
